# Potential application of ultra-low field portable MRI in the ICU to improve CT and MRI access in Canadian hospitals: a multi-center retrospective analysis

**DOI:** 10.3389/fneur.2023.1220091

**Published:** 2023-09-21

**Authors:** Omar Islam, Amy W. Lin, Aditya Bharatha

**Affiliations:** ^1^Department of Neuroradiology, Kingston Health Sciences Centre, Queen’s University, Kingston, ON, Canada; ^2^Department of Medical Imaging, St. Michael’s Hospital, Unity Health Toronto, University of Toronto, Toronto, ON, Canada

**Keywords:** portable MRI, MRI, CT, ICU, radiology

## Abstract

**Background:**

To highlight the value of Portable MRI in ICU and to recommend use case scenarios for portable MRI in ICU patients that may increase capacity for fixed CT and MRI units. Urgent neuroimaging is commonly required in ICU. Typically, ICU patients are transported to Radiology for assessment in fixed CT and MRI units. Portable MRI use in Canadian ICU settings offers the potential advantages of reduced transport risk, earlier diagnosis, improved triaging, as well as the ability to perform frequent re-imaging at the bedside. This frees up time on fixed CT and MRI units, leading to enhanced capacity to perform CT and MRI on other patients. Portable MRI use case scenarios in Canadian institutions have not been established and potential beneficial effect on wait times has not been analyzed.

**Methods:**

A retrospective semi-quantitative descriptive analysis was performed using all ICU neuroimaging requisitions (CT and MRI) over a 12-month period between January and December 2021, at Kingston Health Sciences Centre, Queen’s University (Kingston, Ontario) and St. Michael’s Hospital, Unity Health, University of Toronto (Toronto, Ontario). Indications for portable MRI in ICU patients were established. The number of ICU patients who could potentially undergo portable MRI was determined. Fixed CT and MRI scan times saved were calculated.

**Results:**

In ICU patients, portable MRI could potentially replace fixed CT in 21% and fixed MRI in 26.5% of cases. This equates to annual capacity increase of 1,676 additional patients being able to undergo fixed CT scans and 324 additional patients being able to undergo fixed MRI.

**Conclusion:**

Implementation of portable MRI in the ICU for select neurological indications can have a significant positive impact on CT and MRI wait times in Canadian hospitals.

## Background

Urgent cerebral imaging is commonly required in the Intensive Care Unit (ICU) setting for critically ill patients. Assessment for acute changes in level of consciousness, acute stroke, transient ischemic attack, hydrocephalus, CNS infections, elevated intracranial pressure, and progression of intracranial pathology are some of the common indications for CT and MR imaging in ICU patients (1). The clinical examination of ICU patients is typically not reliable, as most of the patients are intubated, with multiple co-morbidities. There may be superimposed metabolic abnormalities that confound the clinical picture. Patient positioning and lack of ability to move patients also limits proper physical examination (2). Furthermore, in one study of ICU physicians, half of surveyed physicians believed the physical examination was of limited use in the critical care setting (3).

With this backdrop, the importance of neuroimaging cannot be understated (4). For most, if not all ICU patients, neuroimaging is required on an urgent basis to address these relevant clinical questions, and management decisions are predicated on obtaining fast and reliable results of neuroimaging studies. This typically is required within a time frame ranging from minutes to several hours, and is dependant on patient conditions, as well as resource availability in ICU such as physicians, nurses, respiratory technologists, and porters. In the constrained Canadian radiology departments, availability of the CT and MRI scanners is also sometimes a challenge. Despite these challenges, if neuroimaging is required, patients must be transported from the ICU to the radiology department for assessment in traditional (fixed) unit CT and MRI.

Transportation of patients can be complex and is associated with substantial risk. Adverse events related to intra-hospital transfer of critically ill patients can be as high as 60%, with serious adverse events occurring in nearly 10% of transports (5–8). Serious adverse events can include severe hypoxia and hypotension, accidental extubation, and equipment failure (5, 9, 10). Transport of sick ICU patients who may be on ventilators or other life-sustaining devices requires porter and ICU staff availability, including respiratory technologists, nurses, and/or physicians, all of which are typically resource-challenged in a Canadian ICU setting. This issue has been even more evident during the COVID-19 crisis, where nursing shortages and measures for added infection control severely impacted the ability of timely care of ICU patients (11).

Portable MRI (ultra-low field MRI) is a recent technological innovation that allows point-of-care cerebral imaging. The Hyperfine™ portable MRI is an FDA and Health Canada approved 64 mT scanner that can produce T1, T2, FLAIR, and diffusion weighted images of the brain (12, 13). These standard imaging sequences are the bread and butter of neuroimaging of ICU patients in fixed MRI units, and are complimentary to the standard non-contrast head imaging performed of ICU patients using fixed CT units. The use of this novel portable MRI technology in Canadian ICU settings offers the potential advantages of reduced transports of patients, earlier diagnosis, improved triaging, and the ability to perform frequent re-imaging at the bedside (9).

Wait time management for CT and MRI in Canada is a topical issue. The Canadian healthcare system, for the most part, is government funded, and typically through ministries of health at each provincial level. The two Radiology sites in this study are provincially funded through the Ministry of Health, Government of Ontario for CT and MRI operational hours. Lengthening wait times for healthcare access in Canada can be considered a public emergency due to an under-resourced system. This is relevant to all aspects of patient care, including access to primary care, specialists, laboratory testing, and diagnostic imaging, such CT and MRI. In a recent study conducted by the Fraser Institute, patients experienced significant waiting times for various diagnostic technologies across the provinces. In 2022, Canadians could expect to wait 5.4 weeks for a computed tomography (CT) scan, 10.6 weeks for a magnetic resonance imaging (MRI) scan, and 4.9 weeks for an ultrasound (14).

It is therefore highly relevant if alternate methods of imaging are available in the armamentarium of healthcare professionals in the Canadian system to help alleviate the high demands for fixed unit CT and MRI. Resource intensive ICU patients typically require a significant time period in CT and MRI units for performance of their neuroimaging. These time blocks take away from access to fixed CT and MRI units by other hospital patients or outpatients, further exacerbating wait times. The potential diversion of ICU patients from fixed MRI and CT units frees up time on fixed unit CT and MRI, leading to enhanced flexibility to perform fixed unit CT and MRI on other patients. This can have a positive effect on MRI and CT wait times in Canadian institutions.

In a prospective, nonrandomized, observational study, Portable MRI has been deemed a safe and feasible alternative to neuroimaging in ICU patients (15). No patients or staff experienced any adverse events. Throughout the scanning process, patients remained in their room connected to all necessary lines and mechanical ventilation. There were no inadvertent line disconnections or extubations. Providers were able to immediately review images via the hospital’s EMR and PACS resources, thereby leading to instantaneous imaging results. Critical care physicians and nurses were able to continue with routine care during image acquisition (15).

Portable MRI is excellent for assessing anatomical distortions in the brain, allowing confident assessment of subdural and epidural hematomas, ventricular caliber, and shunt placement. It can be used in the assessment of suspected or confirmed cases of elevated intracranial pressure due to its ability to assess the ventricular system and identify lesions that lead to mass effect and/or herniation.

A working protocol for bedside imaging in the ICU has already been established. In one study of 19 ICU patients, images took 39 min to acquire and the average bedside time from start to finish was 90 min per patient (13).

Large territory cerebral strokes are well visualized using portable MRI on DWI, FLAIR, and T2-weighted images, using the B900 Diffusion-weighted sequence (slice thickness 5.8 mm) and the FLAIR sequence to view cortical signal alteration due to cerebral edema. Small posterior fossa (brainstem and/or cerebellar) strokes are less well assessed on portable MRI due to propensity for artifacts in this area. Sensitivity for assessment of these structures, especially the brainstem, may not be sufficient for confident diagnosis. We therefore suggest limiting stroke assessment to the supratentorial compartment and do not include posterior fossa infarct assessment in the clinical indications for portable MRI.

The potential for portable MRI to complement or potentially replace current neuroimaging workflows is extremely valuable. Portable MRI use case scenarios in Canadian institutions have not been established and potential beneficial effect on wait times has not been analyzed.

## Methods

### Study design

A retrospective semi-quantitative descriptive analysis was performed using all ICU neuroimaging requisitions (CT and MRI) over a 12-month period between January and December 2021, at Kingston Health Sciences Centre, Queen’s University (Kingston, Ontario) and St. Michael’s Hospital, Unity Health, University of Toronto (Toronto, Ontario).

### Data collection and analysis

Each of the ICU CT and MRI requisitions were reviewed for their listed patient history and clinical indications.

We chose clinical indications based on our experience with portable MRI, where portable MRI was best suited to assess patients in an ICU setting, rather than being transported to Radiology (Table 1) for fixed unit CT or MRI. This has been previously assessed in a prospective, nonrandomized, observational study of 19 ICU patients (13). In that study patients selected for neuroimaging with portable MRI were admitted to an ICU and had at least one of the following: (1) unexplained encephalopathy or coma, (2) seizures, (3) focal neurologic deficit, (4) abnormal head CT, or (5) elevated inflammatory markers in the blood or cerebrospinal fluid (CSF). Our proposed clinical indications for portable MRI was based on answers to clinical questions that in our experience could be reliably derived from performing non-contrast portable MRI instead of fixed unit CT and MRI.

**Table 1:** Proposed clinical indications for brain imaging using portable MRI in ICU patients.

Subdural hematoma
Epidural hematoma
Hydrocephalus assessment
Extra ventricular drain placement/shunt assessment
Elevated intracranial pressure
Suspected cerebral (ACA, MCA, PCA) stroke assessment and follow-up

We deliberately chose to not include indications of posterior fossa (cerebellar and brainstem) strokes, as we felt the diffusion weighted imaging on portable MRI was prone to artifacts in the posterior fossa and the slice thickness (5.8 mm) was not conducive for detection of tiny acute infarctions. As portable MRI cannot be performed with intravenous contrast (gadolinium agents), we did not include indications of infection, encephalopathy, metastatic disease, or primary CNS neoplasm assessment, where standard of practice dictates intravenous contrast administration. We also did not include requisitions in which seizure was the clinical indication, as in our opinion, such examinations require the higher resolution of fixed 1.5 or 3 T MRI units for thorough assessment.

Table 1 is list of proposed indications for portable MRI.

Based on these proposed clinical indications, we determined the number of the subset of ICU patients who could potentially undergo portable MRI in ICU instead of fixed CT or MRI in the Radiology department. This number was derived from a match on the clinical indication listed on the ICU requisitions with our proposed clinical indications.

Using this number of ICU patients who could undergo portable MRI, we then calculated the potential time saving on fixed unit CT and MRI based on established ICU patient average time requirements in fixed unit CT and MRI. Fixed unit CT and MRI time requirement was defined as the total number of minutes the fixed units were unavailable for other patient use because of room preparation, actual scan time, and clean up for ICU patients.

### Ethics approval and informed consent

The study (Potential Use of Portable MRI in the Intensive Care Setting to Reduce CT Wait Times in Canadian Hospitals—A Multi-Center Retrospective ICU Analysis) received ethical approval from the Queen’s University Office of Research Ethics Compliance (Reference number: TRAQ #: 6037017). Approval date was 22 September 2022. The Office of Research Ethics Compliance reviewed the Quality Initiative Screening Tool and granted an exemption per TCPS 2 Article 2.5 to seek Research Ethics Board (REB) review for this project (TCPS 2 Article 2.5: Quality assurance and quality improvement studies, program evaluation activities, and performance reviews, or testing within normal educational requirements when used exclusively for assessment, management or improvement purposes, do not constitute research for the purposes of this Policy, and do not fall within the scope of REB review). This initiative was formally reviewed by institutional authorities at Unity Health Toronto and deemed to neither require Research Ethics Board approval nor written informed consent from participants.

## Results

Table 2 shows the expected number of fixed unit Brain MRI scans potentially replaceable by portable MRI in ICU patients.

**Table 2:** Expected number of fixed unit brain MRI scans potentially replaceable by portable MRI in ICU patients.

	Total number of ICU patients undergoing non-contrast fixed Brain MRI (2021)		Expected number of ICU patients eligible for Portable MRI based on proposed clinical indications (Table 1)		Percentage of ICU patients eligible for Portable MRI based on proposed clinical indications (Table 1)	
	KHSC	SMH	KHSC	SMH	KHSC	SMH
January	21	25	9	3	42.9%	12.0%
February	18	22	8	0	44.4%	0.0%
March	20	14	11	3	55.0%	21.4%
April	13	25	3	6	23.1%	24.0%
May	8	21	3	6	37.5%	28.6%
June	7	17	4	3	57.1%	17.6%
July	8	24	3	2	37.5%	8.3%
August	13	17	8	3	61.5%	17.6%
September	14	16	7	0	50.0%	0.0%
October	18	22	7	1	38.9%	4.5%
November	7	20	2	2	28.6%	10.0%
December	10	27	4	2	40.0%	7.4%
Total	157	250	69	31	43.9%	12.4%

Table 3 shows the expected number of fixed unit brain CT scans potentially replaceable by portable MRI in ICU patients.

**Table 3:** Expected number of fixed unit brain CT scans potentially replaceable by portable MRI in ICU patients.

	Total number of ICU patients undergoing non-contrast fixed CT (2021)		Expected number of ICU patients eligible for Portable MRI based on proposed clinical indications (Table 1)		Percentage of ICU patients eligible for Portable MRI based on proposed clinical indications (Table 1)	
	KHSC	SMH	KHSC	SMH	KHSC	SMH
January	75	337	11	100	14.7%	29.7%
February	55	291	13	88	23.6%	30.2%
March	70	336	5	74	7.1%	22.0%
April	58	342	13	81	22.4%	23.7%
May	62	312	10	71	16.1%	22.8%
June	66	341	7	50	10.6%	14.7%
July	96	219	16	60	16.7%	27.4%
August	84	169	13	14	15.5%	8.3%
September	79	141	12	20	15.2%	14.2%
October	101	177	16	39	15.8%	22.0%
November	77	197	15	45	19.5%	22.8%
December	65	232	9	56	13.8%	24.1%
Total	888	3,094	140	698	15.8%	22.6%

Based on the proposed list of clinical indications of portable MRI in ICU settings (Table 1), a small but significant number of brain MRI and CT scans can be performed using portable MRI instead of traditional fixed MRI or CT.

At KHSC, 69 out of 157 non-contrast fixed unit brain MRI scans (43.9%) and 140 out of 888 non-contrast fixed unit brain CT scans (15.8%) can be performed using the portable MRI method in ICU. At SMH, 31 out of 250 non-contrast fixed unit brain MRI scans (12.4%) and 698 out of 3,094 non-contrast fixed unit brain CT scans (22.6%) can be performed using portable MRI in ICU.

Using the combined ICU data from both centers, 100 out of total 407 (24.6%) fixed brain MRI scans and 838 out of 3,982 (21.0%) non-contrast fixed brain CT scans on ICU patients may be eligible to be performed using portable MRI in ICU.

At our institutions, the time commitment in the fixed unit MRI is typically 90 min per ICU patient. It is estimated that for those 100 ICU patients at KHSC and SMH (Table 2) who fulfill the selected indications outlined in Table 1 that are required to undergo brain imaging, the total time commitment on a fixed MRI unit on an annual basis is 9,000 min. This equates to the total time saved on the fixed MRI on an annual basis at the two centers if these ICU patients are scanned using portable MRI rather than being transported to the fixed unit MRI. The average time commitment for a typical out-patient scanned on a fixed MRI unit is typically 30 min. If extrapolated based on the potential fixed MRI time saved by diverting 100 ICU patients using portable MRI, this equates to 300 additional out-patients (9,000 min divided by 30 min per out-patient) that can be scanned in the fixed unit MRI on an annual basis.

At our institutions, the time commitment in the fixed unit CT is typically 30 min per ICU patient. For those 838 ICU patients potentially eligible for Portable MRI and therefore avoiding fixed unit CT (Table 3), the total time commitment saved on a fixed unit CT on an annual basis at KHSC and SMH is 25,140 min. The average time commitment for an out-patient CT scan on a fixed unit CT is typically 15 min. If extrapolated based on the potential fixed unit CT time saved by diverting 838 ICU patients using portable MRI, this equates to 1,676 additional out-patients (25,140 min divided by 15 min per out-patient) that can be scanned in the fixed CT scanner on an annual basis.

Portable MRI is a novel imaging modality that can play a vital role in the diagnosis and management of neurological conditions. Portable MRI can be applied in a select group of ICU patients. Portable MRI contributes to patient safety and allows increased efficiency in ICU resource utilization by eliminating the need for patient transportation to the radiology department. Diverting ICU patients allows time on fixed CT and MRI machines to be freed up for other patients, such as outpatients, thereby reducing wait times.

There is limited experience in Canadian centers for the use of Portable MRI. Currently, Portable MRI machines are in use in Ontario at St. Michael’s Hospital, University of Toronto (since March 2022), and Weeneebayko General Hospital, Weeneebayko Area Health Authority, Moose Factory, Ontario (since November 2021). The authors have some of the most experience in Portable MRI use of Radiologists in Canada, notably A.B. and A.L., Neuroradiologists, University of Toronto, who have interpreted the most Portable MRI studies in Canada, and O.I., Neuroradiologist, Queen’s University, who interpreted the first Portable MRI study in Canada.

While it would be useful to compare that number to the potential increased volume of MRIs made capable by another fixed MRI scanner, this is not possible for the most part at Canadian institutions as implementation of fixed MRI is centrally controlled by the Ministry of Health in Ontario, and similar agencies in other provinces. Fixed MRI require a large financial investment, government funding for operational hours, physical space, and a full complement of MR technologists. All of these requirements are either significantly reduced or non-existent with the implementation of portable MRI in an ICU setting.

Based on the authors collective experience, clinical indications have been proposed for brain imaging performed using portable MRI rather than fixed unit MRI or CT. These indications represent 43.9% and 12.4% of fixed unit MRI brain scans and 15.8 and 22.6% of fixed unit CT brain scans at KHSC and SMH, respectively.

We estimate that 300 additional outpatient MRI scans and 1,676 additional outpatient CT scans can be potentially performed using the 90-min time savings per ICU patient on the fixed unit MRI and 30-min time savings per ICU patient on the fixed unit CT at KHSC and SMH.

This has a beneficial effect on MRI and CT wait times. Applied across Canada, the percentage reduction in MRI and CT wait times can lead to thousands of additional patients being served within the restraints of the existing fixed MRI and CT capacities.

The indications for diverting patients to portable MRI in ICU from fixed unit CT and MRI was identical in both institution ICUs. However, there was variability in the number of patients qualifying for portable MRI between the two sites (15.8% vs. 22.6% for CT and 43.9% vs. 12.4% for MR). Variability was more pronounced for MR than for CT. This is interesting and beyond the scope of this analysis, however likely reasons include differences in patient demographics and physician practice parameters. One explanation could be KHSC ICU has a large acute stroke population as a regional acute stroke center, serving a large geographic area and population catchment in southeast Ontario.

It is noteworthy that in Ontario, portable MRI operation, being a recent technological advancement, was not, until recently, encompassed within the authorized scope of practice for Canadian X-ray technologists or nurses. This circumstance could potentially create reservations about adopting this technology. Particularly in situations where staffing resources are already constrained, the staffing aspect presents a notable obstacle to the widespread utilization of portable MRI.

However, a significant development has transpired recently. The Ontario Association of Medical Radiation Technologists has established as of 2021 that any duly qualified x-ray technologist is eligible to operate a portable MRI device, provided they have received a verbal or written directive from a physician. This ruling effectively allows radiology staff, including those beyond the realm of MR technologists, to actively engage in the operation of portable MRI equipment within the ICU setting. Consequently, this opens the door to the potential for technologist availability around the clock, fostering a continuous service for portable MRI. This development is especially crucial, given that the utilization of portable MRI in the ICU domain does not detrimentally impact the availability of limited human resources among CT or MRI technologist. This permits potentially 24/7 operator availability for portable MRI.

In various other jurisdictions, contingent upon the local regulatory landscape, alternative healthcare professionals, such as ICU nurses, might be empowered to operate these units. This prospect has the potential to enhance institutional efficiencies, possibly streamlining operations and optimizing resource utilization.

Portable MRI examinations typically require a scan time ranging from 40 to 45 min. While this duration might initially appear lengthy, it is important to note that the scanning procedure is notably automated, with sequence acquisitions seamlessly integrated into the imaging protocol. This inherent automation presents a distinct advantage. Once the patient is correctly positioned within the MRI scanner and the scanning process is initiated, the technologist or operator overseeing the procedure can effectively allocate their attention to other tasks. This efficiency in operation enables multitasking while the scan acquisition progresses uninterrupted.

Our analysis focused exclusively on the subgroup of ICU patients for whom a non-contrast study was deemed appropriate. Patients who would necessitate intravenous contrast were deliberately omitted from our analysis. While we recognize that fixed MRI scanners operating at 1.5 or 3 T offer superior image resolutions, it ss essential to emphasize that the indications targeted by portable MRI within the ICU context did not demand such elevated image precision. This holds true for scenarios like the assessment of hydrocephalus or the localization of shunt/drain placements, where exceedingly high image resolution was not a prerequisite to answer the clinical question at hand.

Some limitations of our analysis include the selection of our proposed clinical indications for Portable MRI. The clinical indications were selected based on our experience and comfort level with interpretation of Portable MRI examinations. We chose to limit use to select indications where in our experience Portable MRI may display the greatest most diagnostic utility. However, other users may have different clinical indications for portable MRI application, which will affect the usage.

Another variable which will affect the analysis includes time savings on fixed unit MRI and CT based on local clinical practice. The 90 and 30 min time commitment for ICU patients for fixed unit MRI and CT, respectively, is based upon long-standing practice at our institutions. This time commitment may vary from site to site, leading to different calculations in total time saved if Portable MRI was performed in replacement of fixed unit MRI or CT.

Validation of portable MRI results compared to the gold standard of conventional fixed MRI, either at 1.5 or 3 T, has only recently been published. In 2021, a single center study by Sheth et al. of patients with critical illness in an intensive care setting demonstrated the feasibility of low-field, portable MRI (13). This study of 50 patients was conducted in a setting where only portable MRI was used. There was no fixed MRI performed on these patients as a gold standard. Their findings demonstrated the potential role of portable MRI to obtain neuroimaging in complex clinical care settings (13). This can be used as evidence that portable MRI can be used as the sole imaging modality, and thereby positively affect wait times by diverting patients from the radiology department for fixed scans.

A cost–benefit analysis has not been performed. This could prove useful in further validating the utility of portable MRI in ICU to replace a select number of fixed unit CT and MRI scans.

A potential future analysis could explore the utilization of portable CT in the ICU as an alternative to portable MRI. The authors acknowledge that they currently lack experience with portable CT in their institutions. Nevertheless, it can be hypothesized that portable CT might offer advantages in certain scenarios.

Theoretically, a portion of the ICU neuroimaging scans could be conducted using portable CT scanners. This would be particularly applicable in cases where the primary focus is on evaluating conditions such as hemorrhage, hydrocephalus, or shunt positioning. Portable CT could adequately meet the imaging requirements for these indications. However, in scenarios involving acute stroke assessment, portable CT would fall short compared to MRI. This is primarily due to the superior capability of MRI to perform diffusion sequences, a feature not easily achievable with portable CT. It is essential to recognize that portable MRI would remain the preferred option for situations requiring multiple follow-up scans, as this can help reduce patient radiation exposure.

Nonetheless, in our study, we did not employ portable CT scanning. This was due to the unavailability of such equipment at the tertiary care institutions involved in our analysis. Generally, the lack of accessible portable CT technology prevents its incorporation as a standard of care within Canadian intensive care units for the purpose of neurological condition assessment. Exploring the benefits of portable CT vs. portable MRI within a specific subset of ICU patients requiring neuroimaging would present an intriguing avenue for investigation in future studies.

As Portable MRI spatial resolution improves and resultant radiologist comfort level with Portable MRI interpretation improves, the clinical indications will likely broaden for Portable MRI use in ICU patients, allowing them to forgo imaging in fixed scanners. This is especially true in assessment of posterior fossa pathology. Therefore, the positive impact of Portable MRI on fixed unit MRI and CT capacity to perform outpatient scans and reduce wait times will only increase. For example, current slice thickness for diffusion sequences using Portable MRI is 5.8 mm, compared to 3 or 4 mm on typical fixed unit MRI. Using Portable MRI to help exclude small acute strokes will likely become a standard clinical indication, including in the exclusion of posterior fossa strokes, once spatial resolution approximates that of fixed MRI, including for diffusion-weighted imaging. This will permit ability to detect smaller and smaller infarcts on Portable MRI images. Because of the limitation of spatial resolution as it currently stands, and the relatively poor signal to noise, especially for the assessment of the posterior fossa, we limited our clinical indications for portable MRI in ICU patients to cerebral infarction only. Only those patients with clinical indications of cerebral infarctions were included in the patients who could be diverted to portable MRI. It can be postulated that in the future, indication of portable MRI may expand as spatial resolution improves and patients with posterior fossa pathology may also be diverted from fixed MRI and CT units to portable MRI in ICU.

In Canada, the price of portable MRI systems amounts to several hundred thousand dollars, a cost on par with that of portable CT scanners. A recent study conducted by DesRoche et al. (16) delved into the clinical feasibility and cost evaluation of portable MRI utilization within a remote Northern Canadian locale (1). This investigation was carried out in an area devoid of access to traditional MRI resources, specifically the Weeneebayko Area Health Authority situated in Moose Factory, Ontario, Canada.

The findings from this study indicate that the implementation of portable MRI technology within such a remote setting is indeed viable. Notably, the study revealed substantial cost savings when compared to the utilization of fixed MRI systems. In precise terms, the cost savings amounted to $854,841 based on an estimation of 50 patients undergoing portable MRI examinations over the course of a year. Moreover, the assessment extended over a five-year budget impact analysis, which projected an impressive saving of nearly $8 million dollars.

It is noteworthy to mention that, as of now, to the best of our knowledge no comparable cost analysis of employing portable MRI within an intensive care unit (ICU) setting within a tertiary care environment has been published.

## Conclusion

Portable MRI implementation in the ICU setting is feasible for a select range of neurological indications. At our institutions, diversion of select ICU patients to portable MRI would result in an increased capacity of 300 additional outpatient fixed unit MRI and 1,676 additional outpatient fixed unit CT on an annual basis. Based on the combined analysis performed at 2 Canadian centers, a small but significant number of ICU patients could be diverted from conventional practice of fixed unit MRI and CT to portable MRI. This will have a beneficial effect on wait times at resource constraints sites across Canada.

## Data availability statement

The original contributions presented in the study are included in the article/supplementary material, further inquiries can be directed to the corresponding author.

## Ethics statement

The studies involving humans were approved by Queen’s University Office of Research Ethics Compliance (Reference number: TRAQ #: 6037017). The studies were conducted in accordance with the local legislation and institutional requirements. Written informed consent for participation was not required from the participants or the participants’ legal guardians/next of kin in accordance with the national legislation and institutional requirements.

## Author contributions

All authors contributed to the article and approved the submitted version.
